# Effect of Two Different Pretreatments of Brewers Spent Grain Used as Feed Ingredient on Nutritional, Immunological, and Metabolical Parameters in Gilthead Seabream (*Sparus aurata*)

**DOI:** 10.3390/biology14060585

**Published:** 2025-05-22

**Authors:** Francisca P. Martínez-Antequera, Paula Simó-Mirabet, Verónica de las Heras, Marta Román, Juan Miguel Mancera, Juan Antonio Martos-Sitcha, Francisco J. Moyano

**Affiliations:** 1 Centro de Investigación en Agrosistemas Intensivos Mediterráneos y Biotecnología Agroalimentaria (CIAMBITAL), Departamento de Biología y Geología, Facultad de Ciencias Experimentales, Universidad de Almería, 04120 Almería, Spain; fjmoyano@ual.es; 2 Departamento de Biología, Facultad de Ciencias del Mar y Ambientales, Instituto Universitario de Investigación Marina (INMAR), Campus de Excelencia Internacional del Mar (CEI·MAR), 11510 Cádiz, Spain; paula.simo@uca.es (P.S.-M.); veronica.delasheras@uca.es (V.d.l.H.); juanmiguel.mancera@uca.es (J.M.M.); juanantonio.sitcha@uca.es (J.A.M.-S.); 3 Servicio Central de Investigación en Cultivos Marinos (SCI-CM), Campus de Excelencia Internacional del Mar (CEI·MAR), Universidad de Cádiz, 11510 Puerto Real, Cádiz, Spain; marta.romanarias@alum.uca.es

**Keywords:** aquaculture feeds, brewers spent grain, enzymatic pretreatment, microwave heat pretreatment

## Abstract

Brewer’s spent grain (BSG), the primary byproduct generated by the brewing industry, holds significant potential as an ingredient in aquafeeds. However, its high content of non-starch polysaccharides (NSP) restricts the amount that can be incorporated into fish diets. To address these limitations, various pretreatment methods (physical, chemical, or enzymatic) can be applied prior to its inclusion in feed formulations. The objectives of the present study were (i) to optimize the conditions for enzymatic or microwave pretreatments to enhance the nutritional and functional profile of BSG, (ii) to determine the bioaccessibility and availability of specific nutrients and antioxidant compounds in diets including a high amount of BSG (pretreated or not) using an in vitro assay simulating the digestion of the gilthead seabream (*Sparus aurata*), and (iii) to evaluate the effect of such diet pretreatments on growth, metabolism, intestinal microbiota, and oxidative status in live fish. For this study, three experimental diets were formulated: one containing 20% untreated BSG (C) and two containing the same amount of BSG pretreated either enzymatically (H) or with microwave heating (MW). Each diet was administered *ad libitum* to triplicate groups of 22 juvenile sea bream (mean weight 60 g) over a period of three months. The results indicated that the microwave-treated BSG led to improved growth (0.69 ± 0.02%) and feed efficiency (0.80 ± 0.02 weight gain/total feed intake) and enhanced immune status (alkaline phosphatase activity = 11,811.68 ± 3426.92 U/mg SP), compared to the control diet (SGR = 0.59 ± 0.06%; FE: 0.68 ± 0.03 weight gain/total feed intake; alkaline phosphatase activity = 8590.29 ± 3663.44 U/mg SP). Moreover, fish fed on both pretreated BSG diets exhibited significant differences in metabolic parameters and functional profile of their intestinal microbiota when compared to the control group. Consequently, the findings suggest that the pretreatment of BSG, whether by enzymatic or microwave methods, results in notable differences in its nutritional value and the bioavailability of functional components, which, in turn, have a substantial impact on the growth and metabolism of gilthead seabream.

## Simple Summary

Brewer’s spent grain is a byproduct from beer production that is often discarded, despite containing valuable nutrients for use in aquafeeds. However, its high content of complex fibers makes it difficult for fish to digest. This study aimed to improve the nutritional potential of this ingredient by applying enzymatic or microwave heat pretreatments before its inclusion in fish feed. Three diets were formulated: one with untreated grain and two with pretreated grain, either enzymatically or with microwaves. Juvenile gilthead seabreams were fed these diets over a period of three months. Results showed that the microwave-treated grain led to better growth, more efficient feed use, and improved immune response compared to the untreated control. Additionally, both pretreated diets affected the fish’s metabolism and the composition of beneficial gut bacteria. These findings demonstrate that processing this brewing byproduct can enhance its nutritional value and support healthier, more sustainable fish growth, contributing to more efficient and environmentally friendly aquaculture.

## 1. Introduction

Brewer’s spent grain (BSG) is a significant by-product of the brewing industry, with an annual global production exceeding 7 million tons. This lignocellulosic material, rich in nutrients and bioactive compounds, offers a valuable opportunity for valorization. BSG is characterized by high levels of lignin (12–28% of dry matter) and non-starch polysaccharides (NSP) (30–50% of dry matter), primarily derived from barley husks. The NSP fraction comprises cellulose and hemicellulose in roughly equal proportions, with arabinoxylans as the predominant hemicellulose [1,2]. Protein constitutes a major portion of BSG, with essential amino acids, including lysine (an amino acid often limited in cereal-based diets), being particularly abundant [2]. Furthermore, BSG contains various bioactive compounds, including phenolic compounds, flavonoids, and β-glucans, with recognized health-promoting properties [3,4,5]. To maximize the utilization of BSG, numerous studies have investigated enzyme hydrolysis techniques for the extraction of valuable components such as proteins, sugars, and phenolic compounds, aligning with biorefinery principles [2,6,7,8,9,10,11].

Meanwhile, the aquaculture industry faces increasing pressure to enhance sustainability due to the heavy reliance on fishmeal (FM) and fish oil (FO) in feeds for carnivorous species, which has significant environmental implications. This necessitates the identification and incorporation of alternative, sustainable feed ingredients. BSG, with its abundant nutrient profile and potential for bioactive compound delivery, emerges as a promising candidate. By-products offer a dual advantage: they provide essential macronutrients and can serve as sources of functional compounds that enhance various aspects of fish physiology, including growth, metabolism, and immune function [12,13]. Limited research has specifically focused on developing processing techniques for BSG to optimize its nutritional value for use in aquafeeds. While some studies have explored the positive effects of solid-state fermentation (SSF) in species such as Nile tilapia (*Oreochromis niloticus*) and European seabass (*Dicentrarchus labrax*) [14,15], the majority have centered on various forms of enzyme hydrolysis. For instance, He et al. [16] employed alcalase to hydrolyze BSG, generating a product with over 43% protein content after purification and concentration. This hydrolysate successfully replaced up to 50% of fishmeal (FM) in diets for Pacific white shrimp (*Penaeus vannamei*). Similarly, San Martin et al. [17] optimized an enzyme hydrolysis process using a combination of Celluclast^®^ and Protamex^®^ to enhance the protein digestibility and palatability of BSG. Subsequent feeding trials with rainbow trout (*Oncorhynchus mykiss*; [18,19]) and gilthead seabream (*Sparus aurata*; [20]) demonstrated no adverse effects on growth performance or feed conversion ratio at inclusion levels up to 15%.

Microwave heat pretreatment has garnered considerable attention in the processing of plant and animal feed ingredients due to its potential to enhance nutritional quality and nutrient bioavailability [21,22]. The rapid heating generated by microwaves can disrupt cellular structures, leading to matrix rupture and facilitating the release of intracellular components. Microwave heat pretreatment can also inactivate or reduce antinutritional factors, as evidenced by significant reductions in compounds such as phytic acid, polyphenols, oxalates, and saponins in various plant materials [23]. However, to the best of our knowledge, the impact of microwave heat pretreatment on the nutritional value of BSG has not been specifically investigated. Moreover, there is a lack of research focused on optimizing the enzymatic pretreatment of BSG to enhance the bioavailability of both nutrients and antioxidant compounds.

Based on these considerations, this study aims to determine the potential of either enzymatic or microwave heat pretreatments to increase the nutritional value of brewer’s spent grain (BSG). The study encompassed three phases: (1) Optimization: Determination of optimal conditions for the enzymatic and microwave heat pretreatments of BSG that maximize the bioavailability of key nutrients and antioxidant compounds; (2) In vitro evaluation: Assessment of changes in the potential bioavailability of specific nutrients (amino acids and reducing sugars, as well as antioxidant compounds) in experimental diets containing 200 g/kg of treated and untreated BSG after in vitro digestion, mimicking the conditions present in the digestive tract of gilthead seabream (*Sparus aurata*); (3) In vivo assay: Effect of the same diets in metabolic parameters, intestinal microbiota composition, and oxidative status in live fish. The findings will be discussed within the context of enhancing the established potential of BSG for practical application in sustainable aquaculture feeds.

## 2. Material & Methods

### 2.1. Brewer’s Spent Grain (BSG)

Brewer’s spent grain (BSG) was obtained from a local brewery, Origen (Huércal de Almería, Almería, Spain). Following collection, the BSG was oven-dried at 60 °C for 72 h and subsequently milled to a particle size of 0.5 mm using a laboratory mill. Prior to further analysis, the dried and milled BSG was subjected to a preliminary assessment of total phenolic content and antioxidant capacity employing established methodologies [24,25]. Furthermore, the proximate and mineral composition of the BSG was determined by accredited laboratories: KUDAM Laboratory (Alicante, Spain) and Central Research Services at the University of Almería (Almería, Spain), utilizing standard analytical procedures.

### 2.2. Experiment 1: Optimization of BSG Pretreatments

This preliminary experiment focused on optimizing the conditions for enzymatic and thermal pretreatments applied to BSG that maximize the potential bioavailability of protein, carbohydrates, and antioxidant compounds. These conditions should be applied for the pretreatment of BSG used in subsequent experimental phases.

#### 2.2.1. Optimization of Enzymatic Pretreatment

The multienzyme complex used in this pretreatment was a mixture of xylanases, glucanases, arabinofuranosidases, and phytase (Rovabio^®^, Adisseo, Antony, France). A central composite face-centered factorial design (CCFD) [26,27] was used to evaluate the effects of incubation time (range: 6–9 h), humidity (range: 50–70%), and the dose of a commercial enzyme complex (range: 0.4–0.8 mL/kg) on the release of pentoses resulting from NSP hydrolysis and the retention of antioxidant capacity in BSG samples. These ranges were selected based on results from previous experiments involving the enzymatic hydrolysis of BSG using the same enzyme complex [28]. The combination of these factors resulted in a set of 30 different experimental runs, as detailed in Table 1. The design included 13 unique pretreatment combinations, with six replicates of the central point (7.5 h, 60%, 0.6 mL/kg) to estimate experimental error and enhance model reliability, while the remaining pretreatments were tested in duplicate. A 10 g portion of BSG, as described in Section 2.1, was mixed with varying volumes of citrate buffer (pH 5.0, 0.1 M) to achieve the required moisture levels, and the mixtures were placed in plastic containers with a maximum capacity of 100 g. The enzyme complex was dissolved in citrate buffer (pH 5.5, 0.1 M) at the required doses, then carefully sprayed onto and mixed with the BSG. The enzymatic reaction was conducted by incubating the mixture at 45 °C for the specified times, with manual stirring every hour to ensure reaction homogeneity. After incubation, the reaction was halted by freezing the samples at −20 °C until further analysis. The result of the enzymatic hydrolysis of NSP was evaluated by measuring the amounts of free pentoses using the phloroglucinol method, as described by Douglas [29], while the effect on Trolox equivalent antioxidant capacity (TEAC) was assessed using the DPPH and ABTS methods [24,30]. To determine the optimal combination of factor levels that yielded the highest pentose production (used as indicator of NSP hydrolysis) and greatest retention of antioxidant compounds, a composite desirability (CD) function was employed.

#### 2.2.2. Optimization of Microwave Heat Pretreatment

The microwave heat pretreatment (MW) was performed using a commercial microwave oven (Bifinett, KH 1106, Kompernass GmbH, Bochum, Germany) set at a fixed power of 800 W, chosen based on previous studies involving materials with similar compositions [21,22,23]. Two factors were considered in the pretreatment: irradiation time and humidity. A simple factorial design (2 × 3) was employed, considering the following values: 1, 2, and 3 min of irradiation and 50% or 60% of humidity. This resulted in a total of six experimental combinations that were run in duplicate. Each pretreatment was applied to 100 g samples of BSG mixed with distilled water to achieve a moist mass that was then spread out to form a thin cake of 0.5 cm thickness to ensure even heat distribution during irradiation. The samples were placed on a microwave-safe dish with a maximum diameter of 25 cm. After microwave heat pretreatment, the samples were rapidly cooled to 4 °C and stored until further analysis. The release of pentoses and Trolox equivalent antioxidant capacity (TEAC) were measured, as described earlier in Section 2.2.1.

#### 2.2.3. Ingredients and Experimental Diets

Three experimental diets were formulated for use in both the in vitro and in vivo assays. One diet, which included 20% untreated BSG, served as the control (C). The other two diets contained the same amount of BSG, either subjected to enzymatic (H) or microwave heat pretreatment (MW) under the optimal conditions determined in the above-described experiments. The diets were prepared at the Service of Experimental Diets, located at the University of Almeria, Spain (CEIMAR), using a laboratory-scale extrusion machine with a 3 mm die. After preparation, the feeds were dried and stored at 4 °C until use. The formulation and proximate composition of the diets are detailed in Table 2. Prior to their use in the experiments, samples of each feed were analyzed to assess the impact of any of the pretreatments on the potential bioavailability of different key nutrients and bioactive compounds. Soluble protein, reducing sugars, and free pentoses were analyzed in samples of an aqueous extract prepared using 1 g feed in 4 mL of distilled water and then adjusting the pH to 8.0 prior to centrifugation at 4000× *g* for 10 min. Soluble protein was quantified using the Bradford method [31] and bovine serum albumin as the reference standard. For the determination of reducing sugars and pentoses, the aqueous extract was mixed with 96% ethanol at a 1:6 v/v ratio, followed by an additional centrifugation at 4000× *g* for 15 min. Reducing sugars were quantified in the supernatant using the 3,5-dinitrosalicylic acid (DNS) method, as described by Miller [32], while pentoses were measured using the phloroglucinol method [29]. For determination of total phenolics and antioxidant capacity, a 0.5 g sample was suspended in a 1:1 ethanol:water mixture at a 1:10 w/v ratio and maintained at 60 °C for 3 h, with manual homogenization every 30 min. After centrifugation at 4000× *g* for 15 min, total phenolics compounds were quantified in the supernatant following the method outlined by Graça et al. [25] and Trolox equivalent antioxidant capacity (TEAC), as described in Section 2.2.1. All analyses were run in triplicate.

### 2.3. Experiment 2: In Vitro Digestibility Assays

The enzyme extracts required for the in vitro assays were prepared by the mechanical homogenization of tissues obtained after dissection from two different sections (stomach and proximal intestine) of 20 individuals of *S. aurata* weighing about 50 g. The extracts were prepared in cold distilled water (1:10 w/v) followed by centrifugation (14,000× *g*; 15 min, 4 °C) and separation of clear supernatants. The supernatant was then filtered through a dialysis system with a MWCO of 10 kDa (Pellicon XL, Millipore, Billerica, MA, USA), and concentrated extracts were freeze-dried until being required for the assay. After this process, the activities of stomach and intestinal proteases were determined in the extracts; acid protease was measured at pH 2.5 using hemoglobin as substrate [33] while alkaline protease was measured at pH 8.5 by means of casein [34]. The values of protease activities were used as indicators to estimate the amount of extracts required to provide physiological enzyme:substrate ratios in the assays developed for each species. These were calculated considering, on the one hand, the average total production of enzyme measured in several fish in relation to their live weight and, on the other, the average intake per meal of fish of such size, obtained from commercial ration tables.

The assays were carried out using the routine protocol developed by our group, based on the use of two chamber semi-permeable membrane bioreactors [35]. Fish enzyme extracts and diet samples were placed in the upper chamber of the bioreactor and maintained under continuous agitation using a magnetic stirrer. To develop the acid phase of digestion, the upper chamber contained the desired substrate dissolved in water and adjusted to pH 4.5, as well as the crude enzyme extract from the stomach of the selected species while the lower chamber contained distilled water. During the alkaline phase, the pH of the upper chamber was raised to 8.2 using borate buffer (0.1 M supplemented with 20 mM CaCl_2_, sodium taurocholate 45 µM, and NaCl 50 mM) prior to the addition of the intestinal enzyme extracts. The products released during the reaction time and passing across the membrane into the lower chamber were recovered at different time intervals by a constant flow of the same alkaline buffer and used to determine the release of products from the diet samples. The complete arrangement (formed by several experimental units) was maintained within a thermal chamber set at 25 °C. Each diet was tested in triplicate. In addition, the release of products from the feeds in the absence of enzyme hydrolysis was assessed by running assays on which the enzyme extracts were heat-inactivated (placed in a water bath at 100 °C for 5 min). Hydrolysis products (reducing sugars, pentoses, total phenolics, and TEAC) were analyzed using the specific methodologies described in Section 2.2.1 and Section 2.2.2. The release of amino acids was quantified by the orthophthaldehyde (OPA) method described by Church et al. [36].

### 2.4. Experiment 3: In Vivo Assay

The primary objective of this assay was not to develop a practical, growth-maximizing diet but rather to investigate whether or not the chosen pretreatments could significantly improve the nutritional value of BSG compared to its untreated form, with this being evidenced through certain indicative parameters. In this sense, it was presumed that an increased bioavailability of carbohydrates coming from NSP should provide new substrates for some species of intestinal microbiota, and this could be evidenced through modifications in their functional profile. In parallel, an increased bioavailability of antioxidant compounds should have an impact on some indicators of antioxidant status.

#### 2.4.1. Fish and Feeding

A total of 198 gilthead seabream (*S. aurata*) juveniles, with an average body mass of 60.49 ± 0.26 g, were obtained from the Servicios Centrales de Investigación en Cultivos Marinos (SCI-CM, CASEM, University of Cádiz, Puerto Real, Cádiz, Spain). The facility is registered under the Spanish Operational Code REGA ES11028000312 for the breeding, supply, and use of experimental animals. All procedures involving fish maintenance and handling were conducted following the ethical guidelines for experimental procedures in animal research, as approved by the Ethics and Animal Welfare Committee of the University of Cádiz, in compliance with Spanish (RD53/2013) and European Union (2010/63/UE) legislation. The experiments received approval from the Ethical Committee of the Autonomous Andalusian Government (Junta de Andalucía, reference number 15/09/2021/132). The fish were randomly distributed into nine 400 L tanks, each adjusted to a water volume of 330 L, with 22 fish per tank, resulting in an initial stocking density of 4.0 kg/m^3^. The tanks were housed in the SCI-CM experimental facilities under controlled environmental conditions, including a salinity of 37‰, a temperature of 19 °C, and a photoperiod of 10 h of light to 14 h of darkness (10 L:14 D). The three experimental diets (C, H, and MW) were tested in triplicate. The fish were fed daily to visual satiation, with four meals per day, ensuring that all the food provided in each experimental unit was fully consumed. Throughout the feeding trial, which lasted three months (from May to July 2023), no mortality was recorded in any of the experimental groups. Water quality parameters were closely monitored throughout the trial. Temperature, dissolved oxygen, and survival rates were checked daily, while levels of ammonium, nitrite, and salinity were measured weekly to ensure optimal conditions for the fish.

#### 2.4.2. Growth Performance and Biometric Parameters

At the end of the experiment, the following growth parameters and organosomatic indices were evaluated:

(i) Feed Efficiency (FE) = weight gain/total feed intake
(ii) Specific Growth Rate (SGR) = 100 × (ln final body weight − ln initial body weight)/days
(iii) Condition factor (K) = (100 × body weight)/fork length^3^
(iv) Hepatosomatic Index (HSI) = (100 × liver weight)/fish weight
(v) Mesenteric fat Index (MSI) = (100 × mesenteric fat weight)/fish weight
(vi) Intestine Length Index (ILI) = (100 × intestine length)/standard body length

#### 2.4.3. Samplings

At the end of the experimental period, overnight fasted fish (five fish per tank, fifteen per experimental condition) were randomly sampled and deeply anaesthetized with 2-phenoxyethanol in a lethal dose (1 mL/L sea water) prior to obtaining samples of skin mucus, blood, and tissues. Mucus samples were taken by scraping on both dorsal-lateral sides of each fish. Blood was drawn from the caudal vein with heparinized syringes and centrifuged at 3000× *g* for 5 min at 4 °C to separate plasma, which was then snap-frozen in liquid nitrogen and stored at −80 °C until used for further analysis. After this, five fish per tank were cervically sectioned to obtain biopsies of different tissues and were snap-frozen in liquid nitrogen and stored at −80 °C for subsequent biochemical analysis. Samples from the distal intestine (the section between the distal end of the midgut and the anus) were taken, suspended, and homogenized in sterile saline solution and diluted up to 10^−3^ prior to being used in the evaluation of microbial functional diversity.

#### 2.4.4. Functional Diversity of Cultivable Gut Microbiota

Functional biodiversity of cultivable gut microbiota was evaluated in five samples per diet, each one being a pool of three individuals (15 individuals per diet). Assays were carried out using Biolog EcoPlate™ microplates (Biolog, Hayward, CA, USA) according to Feigl et al. [37] with some modifications. One hundred fifty µL of the diluted samples were pipetted into each well of the Biolog EcoPlate™ and incubated at 30 °C for 120 h. After incubation, the optical density (OD) at 590 nm was determined using Gen5 2.04 software by Cytation (Biotek, Winooski, VT, USA). The ODi values and number of substrates were used to calculate the following:

(i) Functional activity intensity as Average Well Color Development (AWCD = ΣODi/N)
(ii) Functional biodiversity as Shannon index (H’ = −Σpi ln(pi), where pi = ODi/ΣODi)
(iii) Functional richness (R = sum of the number of cells where ODi > 0.15)

The results were also expressed as substrate average well color development (SAWCD) for each of the substrate categories [38].

#### 2.4.5. Biochemical Parameters and Stress-Related Markers

Prior to the analysis of the biochemical parameters in muscle, samples of frozen tissue were homogenized by ultrasonic disruption in 7.5 volume ice-cold 0.6 N perchloric acid, neutralized using 1 M KCO_3_, and centrifuged (30 min at 3220× *g* and 4 °C); the supernatants were then isolated to determine tissue metabolites. Prior to centrifugation, one aliquot was taken for triglycerides analyses. Plasma and muscle glucose, lactate, triglycerides, and total cholesterol were analyzed using commercial kits (Refs. 1001200, 1001330, 1001311, and 41021, respectively; Spinreact, St. Esteve d’en Bas, Girona, Spain), adapted to 96-well microplates. Plasma total protein concentration was determined with a BCA Protein Assay Kit (Ref. 23225; Thermo Fisher Scientific Pierce, Waltham, MA, USA) using bovine serum albumin (BSA) protein as the standard. Glycogen concentration was quantified in muscle homogenates using the method described by Decker and Keppler [39], where glucose obtained after glycogen breakdown with amyloglucosidase (Ref. A7420; Sigma-Aldrich, St. Louis, MO, USA) was determined using the commercial kit described above. Plasma cortisol levels were measured with the commercial Cortisol Enzyme Immunoassay Kit (Ref. K003-H1W; Arbor Assays, Ann Arbor, MI, USA) according to the manufacturer’s indications. All assays were performed using a PowerWaveTM 340 microplate spectrophotometer (Bio-Tek Instruments, Winooski, VT, USA) and the Gen5 data analysis software (Bio-Tek Instruments, Winooski, VT, USA) for Microsoft^®^.

#### 2.4.6. Oxidative and Immune Status

The potential variations in the immunological status were assessed by measuring alkaline phosphatase activity in fish skin mucus. Alkaline phosphatase activity was determined using 4-Methylumbelliferyl phosphate disodium salt (Ref. M8168; Sigma-Aldrich, St. Louis, MO, USA) as the substrate following the method described by Fernley and Walker [40]. One unit of activity was defined as 10^3^ RFU. The tissue samples were homogenized (1:10, w/v) in 100 mM phosphate-buffered saline (pH 7.4) at 4 °C using a mini handheld homogenizer (Ref. MT-13K; Hangzhou Miu Instruments Co., Ltd., Hangzhou, China) for 1 min. Different enzyme activities indicative of oxidative status were evaluated in liver homogenates obtained after centrifugation (12,000× *g* for 15 min at 4 °C) by using commercial kits; superoxide dismutase (SOD; Ref. CS0009; Sigma-Aldrich, St. Louis, MO, USA), catalase (CAT; Ref. EIACATC; Thermo Fisher Scientific, Waltham, MA, USA), and glutathione peroxidase (GPx; Ref. 703102; Cayman Chemical, Ann Arbor, MI, USA).

### 2.5. Statistical Analysis

The design and evaluation of the results of the factorial experiments were carried out using the DOE module of Minitab 17 software (Minitab Inc., State College, PA, USA). For the rest of the data (nutrient contents, zootechnical parameters, microbial functional diversity, biochemical and immunological parameters), after evaluation of their normality and homoscedasticity using the Shapiro–Wilk test and the Brown–Forsythe test, respectively, they were analyzed using one-way ANOVA followed by Fisher’s LSD test when appropriate. The 95% confidence level was used for all analyses. When required, data expressed in percentage were previously arc-sin transformed. Those latter analyses were performed using the software Statgraphics Centurion 18 (Statgraphics Corp. CA. EE.UU.) for Windows.

## 3. Results

### 3.1. Experiment 1: Optimization of Pretreatments in BSG

**Figure 1:**
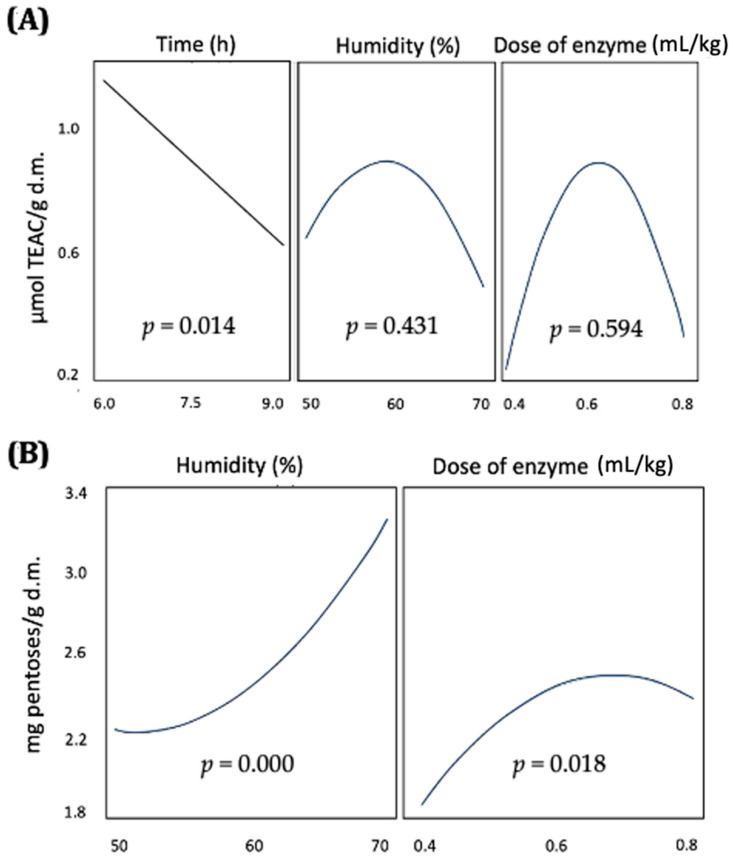
Plot of the main effects evaluated on the release of (**A**) antioxidant capacity and (**B**) pentoses in the optimization of the enzymatic pretreatment using a DOE design.

**Figure 2:**
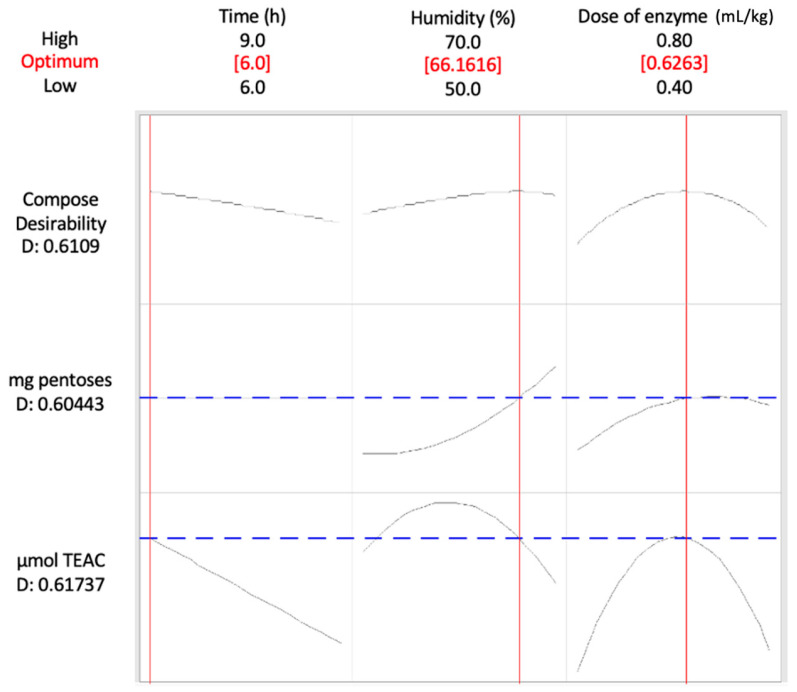
Optimization plot for the maximum release of both pentoses and total antioxidant compounds from BSG under changing conditions of the three factors considered. The optimal solution is noted by a red line. Predicted responses and simple desirabilities are represented by dashed blue lines.

The optimization results of the enzymatic hydrolysis of BSG are presented in Figure 1. The total antioxidant capacity (TEAC) was differentially influenced by the three factors evaluated. TEAC (Figure 1A) decreased rapidly with incubation time, whereas the other two factors, i.e., humidity and enzyme dose, exhibited a quadratic response, with optimal values identified at 60% humidity and 0.6 mL/kg dry weight (d.w.) of enzyme dose. In contrast, the release of pentoses due to carbohydrate hydrolysis (Figure 1B) was significantly affected by humidity in a positive manner and by the enzyme dose, the latter also showing a quadratic response with a peak value at 0.73 mL/kg d.w. The desirability function was employed to simultaneously optimize the factors influencing both pentose release and the stability of antioxidant compounds in BSG (Figure 2). The optimal conditions were determined to be a short reaction time of 6.0 h, relatively high humidity at 66%, and a moderate enzyme dose of 0.63 mL/kg d.w. Under these conditions, pentose hydrolysis was expected to reach 60%, while TEAC should retain 62% of the maximum values observed within the studied ranges.

**Figure 3:**
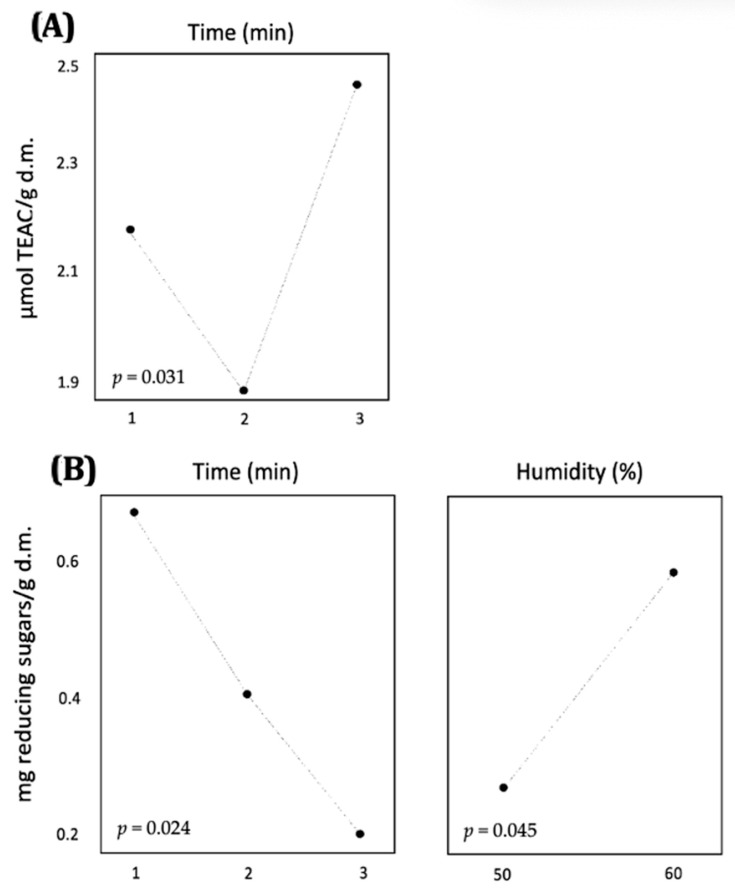
Plot of the main effects evaluated on the release of (**A**) antioxidant capacity and (**B**) reducing sugars in the optimization of the microwave heat pretreatment using a factorial design. d.m.: dry matter.

The effects of the factors considered in the optimization of microwave heat pretreatment are illustrated in Figure 3. Interestingly, TEAC exhibited a two-peak profile, with a minimum value observed after 2 min of pretreatment (Figure 3A). In contrast, the release of pentoses decreased with longer incubation times and was enhanced by higher sample moisture (Figure 3B). Based on these findings, the conditions selected for the pretreatment of BSG in subsequent experiments were 1 min of exposure, 60% humidity, and 800 W power.

### 3.2. Experiment 2: In Vitro Digestibility Assays

The preliminary characterization of the experimental feeds, including components such as soluble protein, reducing sugars, and polyphenols, is presented in Table 3. The inclusion of enzyme-treated BSG (H) in the feed significantly increased the levels of pentoses and total polyphenols. Conversely, the inclusion of microwave-treated BSG (MW) resulted in a significant reduction in soluble protein content but enhanced the release of antioxidant compounds. These differences were largely reflected in the results of the in vitro hydrolysis assays (Table 4). Both pretreatments led to a significant reduction in the amount of potentially bioavailable amino acids compared to untreated BSG. However, the enzyme-treated BSG (feed H) showed a significant increase in the release of pentoses, while the microwave-treated BSG (feed MW) exhibited a similar effect with reducing sugars. Additionally, a significant enhancement in the bioavailability of antioxidant compounds was observed in the H feed, presenting the MW feed intermediate values.

### 3.3. Experiment 3: In Vivo Assay

Although the primary objective of this study was not to develop a practical, growth-maximizing diet but rather to investigate whether or not the chosen pretreatments could significantly improve the nutritional value of BSG compared to its untreated form, evaluation of some zootechnical parameters was carried out. The results of growth performance and biometric indices measured in fish after 3 months of receiving the experimental diets are presented in Table 5. This long period was required to obtain a significant growth due to the use of nutritionally limited diets. The inclusion of enzyme-treated BSG showed no significant effects in any of the parameters calculated. However, fish in the MW group exhibited higher final body mass concomitantly observed with a significantly improved specific growth rate (SGR) and feed efficiency (FE) compared to those fed the control diet. Additionally, fish fed the MW diet also showed a significant increase in intestinal length (ILI).

**Figure 4:**
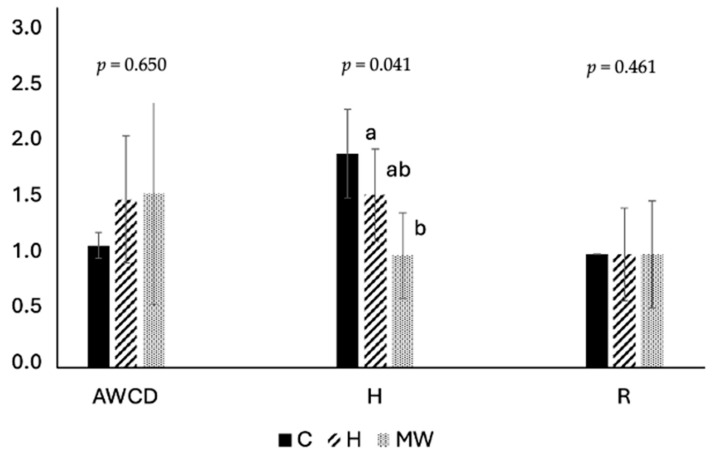
Variations in functional biodiversity of gut microbiota in fish fed on the different experimental diets (C: control; H: enzymatic pretreatment; MW: microwave heat pretreatment). Values not sharing a common letter differ significantly with *p* < 0.05. AWCD: average color development is an index of the total bioactivity; H: Shannon index. (H) is the functional biodiversity index; R: functional richness.

**Figure 5:**
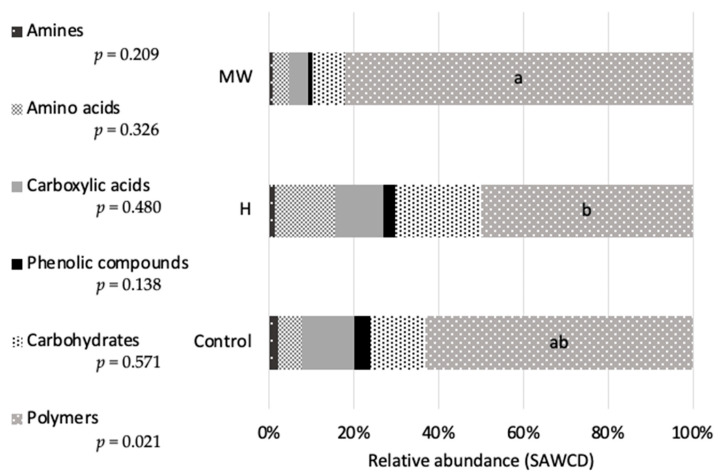
Preferred use of the different types of metabolic substrates by the aerobic intestinal microbiota of fish fed on the experimental diets (C: control; H: enzymatic pretreatment; MW: microwave heat pretreatment) expressed as relative abundance (SAWCD). Values not sharing a common letter are significantly different with *p* < 0.05.

The impact of the diets on the functional profiles of intestinal cultivable microbiota is summarized in Figure 4 and Figure 5. Thermal pretreatment of BSG (MW) had a significant effect on the microbial profile, leading to a reduction in the number of functional groups (lower biodiversity) compared to fish fed the control diet (Figure 4). Additionally, notable differences were found among the dietary treatments regarding the substrates preferred by the microbial populations. The profile in fish fed the H diet displayed distinct characteristics when compared to that of fish receiving the MW diet (Figure 5).

The biochemical and immunological parameters assessed in plasma, muscle, and mucus of fish fed the different experimental diets are detailed in Table 6. Fish fed the H and MW diets exhibited significantly lower plasma lactate levels compared to those on the control diet. Additionally, the H group showed significantly lower levels of plasma cholesterol and protein. Fish on the MW diet also had significantly reduced muscle TAG levels relative to the control diet. Regarding immunological status in the skin mucus, both the H and MW diets significantly increased alkaline phosphatase activity.

The values of the different parameters used to assess oxidative status in fish are presented in Table 7. Fish fed the pretreatment diets (H and MW) exhibited a significant reduction in CAT and GPx levels.

## 4. Discussion

Biotechnological pretreatments for non-conventional ingredients require optimization to ensure the maximum bioaccessibility and bioavailability of nutrients and functional compounds. The results from this study indicate that the factors affecting enzyme activity differentially influence the release of antioxidant compounds and the products of carbohydrate hydrolysis. The assays were conducted at a fixed pH of 5.5, which is optimal for the enzyme activity present in the complex. Under these conditions, the release of pentoses from carbohydrate hydrolysis was significantly and positively influenced by both humidity and enzyme dose. These optimal conditions are consistent with those reported by San Martin et al. [17] for other commercial enzymes, involving a temperature of 55 °C, pH 6, and a fixed solid-to-liquid ratio of 1:1 with water. It is important to note that the conditions used in this enzymatic pretreatment differ considerably from those employed when the goal is to maximize carbohydrate hydrolysis or phenolic extraction from BSG in a biorefinery context [7,41]. In such scenarios, more extreme conditions are typically used, resulting in higher total consumption of time, energy, and enzyme products. Nevertheless, the conditions determined in the present study, using the desirability function, represent a compromise between optimizing carbohydrate hydrolysis and preserving high levels of antioxidant compounds. These conditions included a short reaction time of 6 h, a relatively high humidity of 66%, and a moderate enzyme dose of 0.63 mL/kg dry weight.

Regarding the use of microwave heat pretreatment, the results indicated that it altered the structure of BSG in a way that led to different nutrient and bioactive compound releases compared to enzyme hydrolysis. Microwave heat pretreatment has recently emerged as a promising strategy for enhancing the nutritional value and digestibility of various nutrients in feeds. However, the mechanisms by which microwaves modify ingredient composition are diverse and complex. Microwaves generate heat rapidly and uniformly within materials, which can lead to the disintegration of cellular structures and the rupture of cell walls [42]. This process facilitates the release of nutrients trapped in the ingredient matrix, thereby improving their bioavailability for digestion. Additionally, microwaves can exert non-thermal effects, such as aligning polar bonds and accelerating chemical, biological, and physical processes [43].

Given their potential, numerous studies have focused on maximizing the extraction of compounds from BSG utilizing microwave heat pretreatment. Effective conditions for extracting bioactive compounds typically involve brief treatment durations of less than 10 min [44], the predominant use of water as the solvent [45], high solvent-to-solid ratios approaching 90% [44,45], and power levels not exceeding 1000 W [45,46,47]. Conditions for optimizing carbohydrate hydrolysis also encompass diverse ranges of these parameters. Previous research has demonstrated the positive influence of microwave irradiation on carbohydrate bioavailability in various feed ingredients [48,49,50,51,52]. López-Linares et al. [53] achieved high sugar yields from BSG using temperatures exceeding 190 °C and employing acidic and alkaline solvents. However, as observed with enzymatic pretreatment, the conditions described in these studies were not primarily intended to enhance the nutritional value of BSG as a feed ingredient but rather aimed at maximizing the yields of various compounds. The objective of the present study diverged from this, focusing on optimizing the simultaneous release of both sugars and antioxidant compounds. Consequently, milder conditions were employed, and the utilization of a factorial design facilitated the identification of the optimal combination of time and moisture content for achieving the maximum release of these compounds.

### 4.1. In Vitro Digestibility Assays

In vitro digestive simulations of aquatic animals have been employed for various purposes, such as evaluating the protein quality of feed ingredients [54,55], examining factors that influence the efficiency of enzymatic hydrolysis during digestion [56], or assessing the impact of digestive biochemistry on toxic compounds [57]. However, limited research has investigated antioxidant capacity through in vitro digestive simulation [58]. This area of research holds particular significance, as the antioxidant capacity of ingredients can exhibit substantial variability across different stages of digestion and among individuals [59]. In the present study, in vitro assays revealed that pretreatment with either enzymes or microwave irradiation significantly enhanced the potential bioavailability of carbohydrates and antioxidant compounds. Furthermore, the total antioxidant capacity measured within digestates was influenced not only by the overall digestion time and the specific stage of digestion but also by the type of BSG pretreatment employed. This finding aligns with the results obtained during the optimization of BSG-derived products: pretreatments that increased bioaccessibility also resulted in greater availability of antioxidants in the in vitro digestive simulation.

### 4.2. In Vivo Assay

As outlined in the Introduction, the primary objective of this study was not to formulate a practical diet incorporating BSG to maximize seabream growth. Indeed, a previous study by Estévez et al. [20] demonstrated that incorporating 20% BSG, regardless of whether it was simply dried or enzymatically hydrolyzed, significantly impaired seabream growth and feed digestibility due to its high fiber content. Similar limitations have been observed in other species, such as *Oreochromis niloticus* and *Icatalurus punctatus*, where high inclusion levels of BSG have been shown to reduce growth performance [60]. Nevertheless, this high inclusion level was selected for the present study to investigate whether or not different pretreatments could positively influence the nutritional value of BSG. Despite the anticipated compromise in fish growth performance compared to a standard diet, notable differences emerged between the two processing methods tested. The results confirmed suboptimal growth rates in the fish but also revealed significant disparities between the two pretreatment methods. While the enzymatically treated feed (H) exhibited an improvement in nutritional value, no significant effects were observed on the zootechnical parameters of fish fed this diet. Conversely, fish fed the MW diet demonstrated a significant enhancement in feed efficiency (FE) and specific growth rate (SGR). These findings suggest that microwave heat pretreatment holds considerable promise for enhancing growth performance in animal feed [21,22].

Surprisingly, the in vivo experimental outcomes did not fully corroborate the findings of the in vitro assays. In vitro, the MW diet demonstrated significantly enhanced bioavailability of sugars but exhibited a diminished bioavailability of amino acids compared to the control. This apparent contradiction may be explained by the thermally induced degradation of some amino acids during microwave heat pretreatment, a phenomenon previously reported in studies on heat-processed protein sources [61]. Nonetheless, the increased release of reducing sugars could have compensated for this loss by providing an additional readily available energy source, thereby supporting growth performance [62]. Furthermore, the observed improvement in nutrient absorption, as evidenced by the increased intestinal length in the MW group (Table 5), could also contribute to the superior growth observed in fish fed the MW diet. However, further investigations are warranted to confirm this hypothesis and fully elucidate the underlying mechanisms.

As previously noted, a significant increase of approximately 18% in the intestinal length index (ILI) was observed in fish fed the MW diet compared to those receiving untreated BSG (C). This enhancement in ILI may be associated with the significant reduction in intestinal microbial diversity also observed in fish fed the MW diet. While it is well-established that dietary treatments can significantly influence the intestinal microbiota of aquaculture species [63], there is currently a dearth of research specifically investigating the impact of microwave heat pretreatment on this aspect. Microwave heat pretreatment of BSG has been demonstrated to increase the bioavailability of both reducing sugars and phenolic compounds within the intestinal milieu. Both of these factors could exert a significant influence on the microbial profile. Villasante et al. [64] reported that carbohydrate-rich diets for Atlantic salmon resulted in a reduction in microbial diversity while simultaneously improving diet utilization. Similarly, Gericke et al. [65] observed that enzymatically treated BSG incorporated into the diet of African catfish significantly reduced microbial diversity within the intestinal tract.

Furthermore, dietary polyphenols are well-known to exert a significant influence on the diversity and functional activity of the gut microbiota through both direct and indirect interactions [66,67]. Polyphenols can stimulate or inhibit bacterial growth, while the gastrointestinal microbiota is capable of metabolizing these compounds. Conversely, polyphenols can significantly alter the composition, diversity, and metabolic activity of intestinal bacterial communities [68]. A reduction in microbial diversity can be associated with a diminished intestinal absorptive capacity. Li et al. [69] demonstrated in rhesus macaques that the dysbiosis of commensal bacteria induced by antibiotics adversely affects the epithelial cells of the intestinal mucosa. In this context, the observed increase in intestinal size in fish fed the MW diet may represent a physiological compensatory mechanism to counteract the reduced absorptive capacity resulting from alterations in the gut microbiota’s functional profile. This compensatory mechanism exhibits similarities to that described when increasing the dietary proportion of vegetable ingredients in feeds for carnivorous species [70]. Although variations in the composition of microbial populations in fish fed after MW pretreatment can only be presumed, as they were not directly assessed using a genomic approach, some indirect effects may still be considered. Among these, it cannot be entirely discounted that the reduction in microbial diversity may reflect the predominance of bacterial groups that effectively utilize the substrates whose bioavailability was enhanced by the pretreatments. In this context, such microbial predominance could be related to modifications in intestinal absorptive capacity.

Significantly lower plasma lactate levels were observed in fish fed both enzyme-treated (H) and microwave-treated (MW) BSG diets compared to the control group. This reduction can be attributed to several factors, including decreased lactate production through a reduction in anaerobic metabolism and enhanced lactate clearance. Lactate can be effectively utilized in various organs, such as the liver, for conversion to pyruvate via the Cori cycle [70]. Furthermore, lower plasma lactate levels may also serve as an indicator of more efficient energy metabolism, characterized by a closer alignment between the energy derived from food intake and the physiological demands for growth and other essential metabolic processes, including swimming and immune function [28].

Conversely, the lower plasma protein and cholesterol levels observed in fish fed the enzyme-treated BSG (H) diet may be indicative of enhanced nutrient digestibility and utilization, leading to the greater absorption of dietary proteins and lipids [71]. This suggests that lower circulating levels of these metabolites may be associated with more efficient nutrient utilization for growth and overall fish welfare. While elevated plasma protein levels might reflect increased protein anabolism and a more active metabolic state [72], potentially indicating greater growth potential in fish fed the MW diet or, at the very least, improved feed efficiency, further investigation is warranted to confirm this hypothesis.

Collectively, fish fed the enzyme-treated BSG (H) diet exhibited lower plasma levels of cortisol, cholesterol, and protein, and a tendency towards reduced muscle TAG content, potentially suggesting a less active or reduced metabolic rate compared to fish fed the microwave-treated BSG (MW) diet [72]. These findings underscore the differential effects of the two BSG pretreatments on fish physiology and metabolism.

Significantly reduced levels of triacylglycerols (TAG) were observed in the muscle tissue of fish fed the MW diet, suggesting enhanced lipid bioavailability and utilization within the fish. Previous studies have demonstrated that microwave heat pretreatment can significantly enhance the extraction and digestibility of lipids from various feed ingredients, including BSG. This process potentially increases the availability of fatty acids and triacylglycerols for absorption within the fish’s digestive tract [73]. This improved lipid utilization, reflected by reduced muscle TAG accumulation, has important implications for fish growth, performance, and overall health. Lipids serve as a crucial energy source and play a vital role in numerous physiological processes, including growth, reproduction, and immune function in aquatic organisms [74]. Moreover, reduced muscle TAG levels may also contribute to an extended shelf life of the fillets by minimizing lipid oxidation and enhancing fillet quality, a highly desirable attribute within the aquaculture industry [75].

A significant increase in the activity of mucus alkaline phosphatase was observed in gilthead seabream fed both enzyme-treated (H) and microwave-treated (MW) BSG diets. This finding aligns with the review by Lallès [76], which emphasizes that alkaline phosphatase activity in fish skin mucus is significantly influenced by multiple factors. These include environmental conditions, nutritional status, and dietary supplementation. Lallès [76] concludes that the modulation of alkaline phosphatase activity is a complex process influenced by various factors. This supports the idea that dietary pretreatments, such as the BSG pretreatments used in this study, may positively impact the fish immune response by enhancing enzyme activity in the mucus.

Maintaining elevated levels of alkaline phosphatase activity in skin mucus is essential for optimal fish health. Several studies have shown a strong positive correlation between increased alkaline phosphatase activity and improved fish welfare. This positive association has been substantiated by research investigating dietary interventions such as the inclusion of bioactive compounds or plant extracts [77,78,79,80] as well as the utilization of probiotics and prebiotics [81,82] in aquaculture feeds. Endogenous antioxidant systems, comprising enzymes such as superoxide dismutase (SOD), catalase (CAT), and glutathione peroxidase (GPx), are crucial for cellular defense against oxidative damage. SOD catalyzes the dismutation of superoxide radicals into molecular oxygen and hydrogen peroxide, which are subsequently detoxified by CAT or GPx [83]. The activation of these antioxidant enzymes in response to oxidative challenges, such as increased metabolic activity or environmental stressors, is well-documented [84].

Although this study did not specifically induce oxidative stress, it revealed notable differences in antioxidant enzyme activities. Fish fed the enzyme-treated (H) and microwave-treated (MW) BSG diets exhibited significantly lower levels of catalase (CAT) and glutathione peroxidase (GPx) compared to the control group. These findings suggest that both pretreatments may enhance the bioavailability of antioxidant compounds within BSG [85], thereby potentially mitigating the oxidative stress [86] generated during normal metabolic processes more effectively than untreated BSG. Consequently, this increased antioxidant capacity derived from the diet may result in a reduced reliance on the activity of endogenous antioxidant enzymes, such as CAT and GPx, to maintain cellular redox homeostasis. Such a decrease in antioxidant enzyme activity aligns with a reduction in oxidative stress, as supported by various studies in different organisms [87,88,89].

Furthermore, dietary supplementation with antioxidants, such as alpha-tocopheryl acetate [90] or carotenoids derived from *Dunaliella salina* [91], has been shown to elicit a reduction in hepatic catalase activity in rainbow trout, alongside other notable alterations in antioxidant defense mechanisms, such as an upregulation in the expression of antioxidant genes. Given these findings, future research endeavors should prioritize investigating the effects of maximizing the bioavailability of antioxidant compounds within BSG under conditions that stimulate the production of reactive oxygen species (ROS). This research should not only encompass the assessment of the activity of the aforementioned antioxidant enzymes (SOD, CAT, and GPx) but also encompass the evaluation of Nrf2 (nuclear factor erythroid 2-related factor 2) activation. Nrf2 is a pivotal transcriptional regulator of cellular responses to oxidative stress [92,93].

## 5. Conclusions

The results obtained in the present study demonstrate that the nutritional value of some fibrous by-products, like BSG, with potential application as ingredients in fish feeds, can be improved through an adequate pretreatment. The employment of different types of factorial designs is highly advisable in the selection of the optimal conditions to be used in such pretreatments that maximize the bioaccessibility and potential bioavailability of both nutrients and bioactive compounds. These latter aspects can be evaluated in a cost-effective manner using in vitro assays simulating the digestion of the target fish species. While both enzymatic and microwave heat pretreatments demonstrate positive effects, significantly increasing the availability of carbohydrates (reach 60% within the studied ranges) and antioxidant compounds (reach 62% within the studied ranges), in vivo assays in gilthead seabream (*Sparus aurata*) revealed that microwave heat pretreatment yielded superior results, leading to an improved specific growth rate (SGR = 0.69 ± 0.02%), enhanced feed efficiency (0.80 ± 0.02 weight gain/total feed intake), and a more favorable intestinal microbiota profile (lower biodiversity). These findings suggest that microwave heat pretreatment emerges as a promising and potentially cost-effective strategy for enhancing the nutritional quality of BSG and other similar lignocellulosic feed ingredients (under optimal conditions, pentose hydrolysis is expected to reach 60%, while the TEAC value should retain 62% of the maximum values observed). Potential avenues for future research can explore the effect of combining both enzyme and microwave heat pretreatments in order to increase the nutritional value of this type of ingredient, thereby expanding their potential applications in sustainable aquaculture.

## Figures and Tables

**Table 1:** Central composite face-centered design (CCFD) used to evaluate the effect of several factors on the release of different compounds from the enzyme pretreatment of brewer spent grain.

Runs	Blocks	Time (h)	Moisture (%)	Doses (mL/kg)
1	1	7.5	70	0.4
2	1	9	60	0.4
3	1	6	60	0.4
4	1	9	60	0.8
5	1	7.5	50	0.8
6	1	7.5	70	0.8
7	1	7.5	70	0.8
8	1	6	60	0.8
9	1	6	60	0.8
10	1	9	60	0.8
11	1	7.5	60	0.6
12	1	7.5	60	0.6
13	1	7.5	50	0.4
14	1	6	70	0.6
15	1	7.5	50	0.8
16	1	9	70	0.6
17	1	6	50	0.6
18	1	7.5	60	0.6
19	1	7.5	70	0.4
20	1	9	70	0.6
21	1	6	70	0.6
22	1	9	60	0.4
23	1	9	50	0.6
24	1	6	50	0.6
25	1	6	60	0.4
26	1	7.5	60	0.6
27	1	9	50	0.6
28	1	7.5	60	0.6
29	1	7.5	50	0.4
30	1	7.5	60	0.6

**Table 2:** Ingredients and proximate composition (in g/100 g dry matter, mean values ± SD) of the experimental diets used in the study (C: control; H: enzyme pretreatment; MW: microwave heat pretreatment). BSG: brewer’s spent grain; BSGH: brewer’s spent grain treated with enzymes; BSGMW: brewer’s spent grain treated with; CPSP: hydrolyzed fish protein; NFE; nitrogen-free extract; CF: crude fiber.

Ingredients	C	H	MW
Fish meal LT94	120	120	120
Soycomil 60	220	220	220
Corn gluten meal	90	90	90
Soybean meal 50	50	50	50
Wheat gluten	110	110	110
Fish oil	80	80	80
Rapeseed oil	35	35	35
Wheat bran	70	70	70
BSG	200		
BSGH		200	
BSGMW			200
Lecithin	1	1	1
Methionine	1	1	1
Lysine	1	1	1
Vitamin/mineral premix	10	10	10
Monocalcium phosphate	3	3	3
CPSP	5	5	5
Proximate composition (g/100 g)			
Crude Protein		40.6 ± 0.8	
Total Fat		16.0 ± 0.2	
NFE		30.6 ± 1.4	
CF		3.9 ± 0.6	
Ash		5.0 ± 0.7	
Energy Value (KJ/100 g)		1830 ± 157	

**Table 3:** Contents in some specific nutrients and antioxidant compounds in the experimental feeds (g/100 dry matter). Statistical comparisons between feeds prior to the enzyme and microwave heat pretreatment are detailed in superscript letters. Values not sharing the same letter differ significantly at *p* < 0.05.

Parameter (g/100 g Diet)	C	H	MW	*p*-Value
Soluble protein	3.262 ± 0.177 ^a^	2.932 ± 0.264 ^ab^	2.760 ± 0.094 ^b^	0.032
Reducing sugars	3.036 ± 0.159	3.033 ± 0.242	2.993 ± 0.206	0.457
Pentoses	0.110 ± 0.003 ^a^	0.189 ± 0.011 ^b^	0.110 ± 0.009 ^a^	0.000
Total polyphenols	1.015 ± 0.050 ^a^	1.271 ± 0.076 ^b^	1.136 ± 0.056 ^a^	0.001
µmol TEAC (ABTS)/100 g diet	0.143 ± 0.004 ^a^	0.144 ± 0.003 ^a^	0.152 ± 0.001 ^b^	0.020
µmol TEAC (DPPH)/100 g diet	0.150 ± 0.009	0.154 ± 0.007	0.148 ± 0.007	0.231

C: control; H: enzymatic pretreatment; MW: microwave heat pretreatment.

**Table 4:** Products released after in vitro hydrolysis simulating digestion of gilthead seabream of the experimental diets. Values not sharing a common letter are significantly different with *p* < 0.05.

Total Released	C	H	MW	*p*-Value
mg amino acids	50.98 ± 1.83 ^a^	44.16 ± 0.59 ^b^	44.20 ± 1.20 ^b^	0.003
mg reducing sugars	25.84 ± 0.68 ^a^	27.17 ± 0.76 ^ab^	28.14 ± 0.90 ^b^	0.031
mg pentoses	9.32 ± 0.80 ^a^	10.64 ± 0.30 ^b^	9.42 ± 0.23 ^a^	0.034
µmol TEAC (ABTS)	13.43 ± 1.59 ^a^	16.80 ± 0.04 ^b^	15.05 ± 0.34 ^ab^	0.042

C: control; H: enzymatic pretreatment; MW: microwave heat pretreatment.

**Table 5:** Growth performance and biometric parameters measured in fish fed on the experimental diets. Values are presented as mean ± SD. Values not sharing a common letter are significantly different with *p* < 0.05.

Parameter	C	H	MW	*p*-Value
Initial body mass (g/fish)	60.43 ± 0.54	60.77 ± 0.39	60.27 ± 0.37	0.402
Final body mass (g/fish)	98.44 ± 3.63 ^a^	101.70 ± 2.26 ^ab^	106.72 ± 1.40 ^b^	0.022
FE	0.68 ± 0.03 ^a^	0.73 ± 0.03 ^a^	0.80 ± 0.02 ^b^	0.005
SGR (%)	0.59 ± 0.06 ^a^	0.60 ± 0.03 ^ab^	0.69 ± 0.02 ^b^	0.040
K	1.83 ± 0.16	1.85 ± 0.11	1.89 ± 0.10	0.099
HSI (%)	1.46 ± 0.28	1.26 ± 0.31	1.46 ± 0.35	0.147
MSI (%)	2.16 ± 0.78	1.63 ± 0.62	2.30 ± 0.87	0.091
ILI (%)	69.00 ± 16.06 ^a^	67.59 ± 13.50 ^a^	84.52 ± 4.97 ^b^	0.003

C: control; H: enzymatic pretreatment; MW: microwave heat pretreatment; FE: Feed Efficiency; SGR: Specific Growth Rate; K: condition factor; HSI: Hepatosomatic Index; MSI: Mesenteric fat Index; ILI: Intestine Length Index.

**Table 6:** Biochemical and immunological parameters measured in plasma, muscle, and mucus of fish fed on the different experimental diets. Values are presented as mean ± SD. Values not sharing a common letter differ significantly with *p* < 0.05.

	C	H	MW	*p*-Value
Plasma				
Cortisol (ng/mL)	18.30 ± 20.29	6.11 ± 4.71	14.62 ± 15.50	0.836
Glucose (mM)	4.49 ± 0.35	4.62 ± 0.47	4.58 ± 0.44	0.719
Protein (mg/mL)	36.50 ± 3.48 ^ab^	33.95 ± 2.76 ^b^	38.94 ± 3.34 ^a^	0.001
Cholesterol (mg/dL)	248.04 ± 32.40 ^ab^	202.87 ± 47.58 ^b^	258.13 ± 63.16 ^a^	0.012
Lactate (mM)	1.66 ± 0.24 ^b^	1.27 ± 0.17 ^a^	1.33 ± 0.31 ^a^	0.000
TAG (mM)	2.51 ± 1.24	2.32 ± 0.79	3.01 ± 1.20	0.265
Muscle				
Glucose (mM/g ww)	1.71 ± 0.65	1.76 ± 0.36	1.71 ± 0.56	0.959
Glycogen (mM/g ww)	0.73 ± 0.72	1.03 ± 1.23	0.51 ± 0.48	0.417
Lactate (mM/g ww)	58.59 ± 15.75	57.44 ± 9.26	57.99 ± 7.62	0.965
Cholesterol (mg/dL/g ww)	990.59 ± 599.79	756.71 ± 394.67	602.43 ± 378.61	0.038
TAG (mM/g ww)	24.28 ± 9.46 ^a^	18.13 ± 8.00 ^ab^	17.92 ± 10.41 ^b^	0.836
Mucus				
U alkaline phosphatase/mg SP	8590.29 ± 3663.44 ^a^	12,384.02 ± 4395.94 ^b^	11,811.68 ± 3426.92 ^b^	0.047

C: control; H: enzymatic pretreatment; MW: microwave heat pretreatment.

**Table 7:** Activities of different enzymes indicative of the oxidative status measured in the liver of fish fed on the different experimental diets. Values are presented as mean ± SD. Values not sharing a common letter differ significantly with *p* < 0.05.

Enzymatic Activity	C	H	MW	*p*-Value
U SOD/mg SP	0.97 ± 0.21	1.09 ± 0.27	1.10 ± 0.29	0.431
U CAT/mg SP	9.40 ± 0.96 ^a^	8.57 ± 0.72 ^b^	8.52 ± 0.94 ^b^	0.042
U GPx/mg SP	11.96 ± 2.23 ^a^	10.03 ± 2.14 ^b^	10.84 ± 1.35 ^b^	0.046
picomol MDA/mg SP	449.66 ± 223.63	355.83 ± 80.78	378.08 ± 115.38	0.346

C: control; H: enzymatic pretreatment; MW: microwave heat pretreatment; SOD; superoxide dismutase, CAT; catalase, GPx; glutathione peroxidase.

## Data Availability

Data will be made available on request.
